# Time-frequency time-space long short-term memory networks for image classification of histopathological tissue

**DOI:** 10.1038/s41598-021-93160-5

**Published:** 2021-07-01

**Authors:** Tuan D. Pham

**Affiliations:** Center for Artificial Intelligence, Prince Mohammad Bin Fahd University, Khobar, 31952 Saudi Arabia

**Keywords:** Biological techniques, Mathematics and computing

## Abstract

Image analysis in histopathology provides insights into the microscopic examination of tissue for disease diagnosis, prognosis, and biomarker discovery. Particularly for cancer research, precise classification of histopathological images is the ultimate objective of the image analysis. Here, the time-frequency time-space long short-term memory network (TF-TS LSTM) developed for classification of time series is applied for classifying histopathological images. The deep learning is empowered by the use of sequential time-frequency and time-space features extracted from the images. Furthermore, unlike conventional classification practice, a strategy for class modeling is designed to leverage the learning power of the TF-TS LSTM. Tests on several datasets of histopathological images of haematoxylin-and-eosin and immunohistochemistry stains demonstrate the strong capability of the artificial intelligence (AI)-based approach for producing very accurate classification results. The proposed approach has the potential to be an AI tool for robust classification of histopathological images.

## Introduction

Image analysis in pathology is an important task that helps provide pathologists with quantitative information to be discovered in complex characteristics of pathology images. Conventional pathological quantification, which is based on the expertise of pathologists, is subjective in assessment, time-consuming for the analysis of large volumes of data, and may encounter difficulties when the reproducibility of results is desired. These factors have arisen the need for automated quantification of digital pathology data.

Histology is the study of the microscopic anatomy of biological tissues, while histopathology is a field of histology that involves the study of diseased tissue. The benefits of automated image analysis of histopathological images are multi-fold^1^. Not only from the perspective of the ability for rendering accurate diagnosis, but the automated analysis can also provide insights into disease mechanisms for understanding biological abnormalities, optimal clinical patient-specific treatment, and biomarker discovery.

Automated image analysis of spatial structures of histopathological images were carried out in works reported in^2,3^. A type of advanced machine-learning method such support vector machines (SVM) was utilized to develop a system for classifying normal tissue and tissue lesions from liver, lung, spleen, and kidney of bovine animals into different histologic categories^4^.

As image processing and classification using deep learning has been realized as a major direction of research in medical prognostics and health management^5^, using the state-of-the-art methods in artificial intelligence (AI) for pattern classification, several deep-learning models have recently been used for classification in digital pathology^6,7^. Some of these works include a self-designed convolutional neural network (CNN) model for necrosis detection in whole-slide images of gastric cancer^8^, the use of a CNN model for pathology-based prediction of survival outcome of patients with lung cancer^9^, a pre-trained CNN (Inception v3) for detecting cancer subtype or gene mutations from histopathological images of non-small cell lung cancer^10^, pre-trained CNNs for identifying histologic growth patterns of lung cancer^11^, and Bayesian CNN for classifying histopathological images of colorectal cancer^12^. More recently, fusion of deep-learning features was performed for classifying histopathological images of breast tissue^13^; and texture features extracted from histopathological tissue images of prostate cancer were used for image classification with support vector machines to provide Gleason scores to the patients’ whole slide images, and the results found to be better than the use of deep learning^14^.

In this study, recurrent neural networks that learn time-frequency and time-space features for classification of time series or sequential data^15^ is further developed for classifying histopathological images. The networks receive input as the combination of multiple features extracted in time-frequency and time-space domains of the time series transformed from the images. The class modeling is then designed for training the networks. Comparative results obtained from testing three public datasets of histopathological images, including haematoxylin-and-eosin (H&E) stained tissue images of colorectal cancer, H&E stained tissue images of heart failure, and immunohistochemistry (IHC) stained tissue images of rectal cancer, show the capability of achieving very accurate classification by the current approach.

## Materials and methods

### Image data

Three public databases are used in this study and descibed as follows.

#### H&E colorectal-cancer data

The colorectal-cancer (CRC) histology data used in this study were originally studied in^16^. The H&E stained tissue images of the CRC are publicly available at URL: http://doi.org/10.5281/zenodo.53169. The dataset consists of ten anonymized H&E stained CRC tissue slides. Tumors of both low grade and high grade were included in the dataset. The slides were first digitized, then contiguous tissue areas were manually annotated and tessellated to produce 625 non-overlapping tissue images of $150 \times 150$ pixels for each of 8 types of tissue, resulting in a set of 5000 images. There are 8 tissue types for classification, which are: (1) tumor epithelium (tumor), (2) simple stroma (stroma), (3) complex stroma (complex), (4) immune cells (lymphoid), (5) debris, (6) normal mucosal glands (mucosa), (7) adipose tissue (adipose), and (8) background (no tissue or empty).

**Figure 1:**
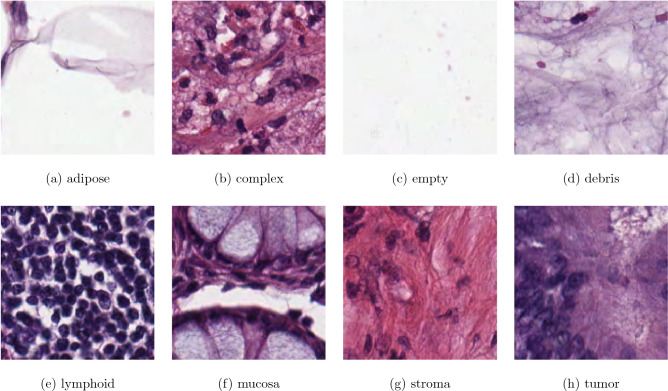
H&E stained tissue images of colorectal cancer.

Figure 1 shows selected samples of the H&E stained CRC tissue images of the dataset.

#### H&E heart-failure data

The H&E stained heart-failure tissue image dataset, which includes left ventricular tissue from 209 patients was originally studied in^17^. The H&E stained tissue images of human heart failure are publicly available at the following URL: https://idr.openmicroscopy.org/webclient. The dataset consists of two cohorts of patients: (1) heart failure (*N* = 94) and (2) without heart failure (*N* = 115). The heart-failure tissue was collected from patients with clinically diagnosed ischemic cardiomyopathy (*N* = 51) or idiopathic dilated cardiomyopathy (*N* = 43). The non-failing patients were organ donors without a history of heart failure. All tissue types were sectioned, stained, and scanned during the data acquisition. The whole slide image of each patient was down sampled to 5$\times$ magnification, where eleven non-overlapping images considered as the regions of interest were extracted and the tissue border was manually refined. The total number of images for the heart-failure and non-heart-failure cohorts are 1034 and 1265, respectively, resulting in a dataset of 2299 images of $250 \times 250$ pixels.

**Figure 2:**
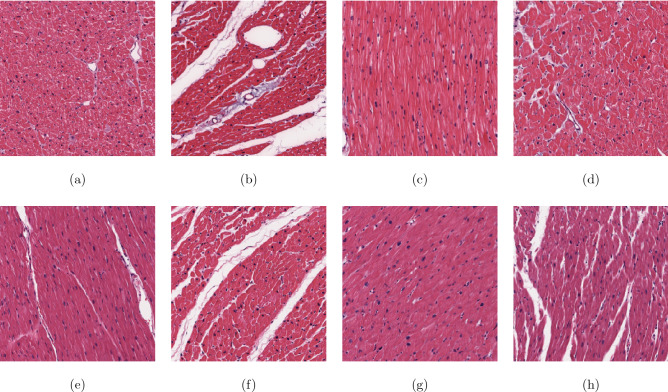
H&E stained heart tissue sections: (**a**)–(**d**) normal condition, and (**e**)–(**h**) failure.

Figure 2 shows selected samples of H&E stained sections of heart-failure and non-heart-failure tissue types.

#### IHC rectal-cancer data

The data were obtained from a cohort of 143 patients with rectal cancer from the Swedish rectal cancer trial of preoperative radiotherapy (pRT) between 1987 and 1990^18^. All patients were performed locally curative resection. Among them, 77 patients received tumor resection alone, and 59 received pRT followed by surgical tumor resection. The pRT was given at a total dose of 25 Gy in 5 fractions over a median of 8 days (6–14 days) before the surgery. The surgical tumor resection was carried out in a median of 4 days (range 0–8 days) after the pRT. Samples were collected from biopsy ($n= 96$), primary cancer or surgically resected tumor ($n=136$), adjacent normal mucosa ($n= 79$), and distant normal mucosa ($n=119$). The distant normal mucosa was taken from proximal or distal margin (4–35 cm from the primary tumor) of the resected rectum and was histologically free from tumor. The adjacent normal mucosa sample was taken adjacent to the primary tumor and was histologically free from tumor. None of the patients received preoperative or adjuvant chemotherapy. The mean follow-up period or the patients was 107 months (range 0–309 months).

The whole dataset has 235 images, where the size for each image is about $2500 \times 2700$ pixels. The image subsets consist of 40 and 14 images of biopsy without pRT having survival rate greater and less than 5 years, respectively; 32 and 11 images of biopsy with pRT having survival rate greater and less than 5 years, respectively; 54 and 25 images of tumor without pRT having survival rate greater and less than 5 years, respectively; and 36 and 23 images of tumor with pRT having survival rate greater and less than 5 years, respectively. Details about the procedure for the image extraction and the publicly available dataset for investigating the prediction and prognosis of DNp73 were described in^19^.

**Figure 3:**
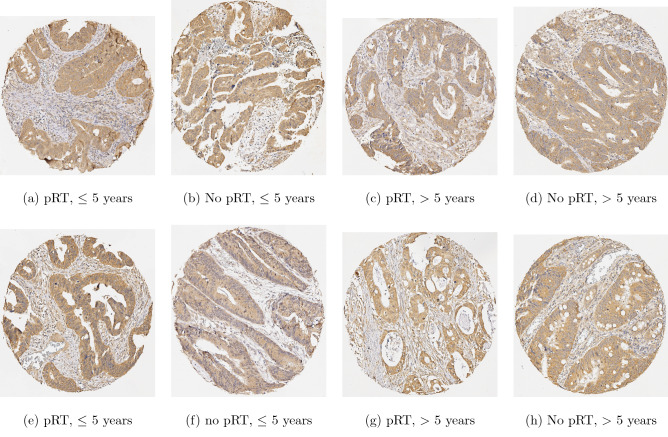
IHC stained tissue images of rectal cancer: (**a**) biopsy, and (**b**) tumor.

Figure 3 shows selected samples of IHC stained images of the rectal cancer obtained for biopsy and primary tumor samples with and without pRT.

### Image vectorization

Because LSTM networks were designed for learning order dependence in time series or sequential data, the conversion of time-independent images into time series is necessary for extracting sequential features that will be used as input into the network. To extract features of the time series of histopathological images in time-frequency and time-space domains, the color (3D) images were first converted into grayscale (2D) images. Using the grayscale images, the vectorization of a 2D image or matrix is a linear transformation that converts the image into a column vector. Specifically, the vectorization of an $M \times N$ image *I*, denoted as *J*, is the $MN \times 1$ column vector obtained by stacking the columns of the image *I* on top of one another, giving

1 $$\begin{aligned} J = [I_{11}, \dots , I_{M1}, I_{12}, \dots , _{M2}, I_{1N}, \dots , I_{MN}]^T. \end{aligned}$$

Thus, *J* results in a time series of image *I*, which is ready for feature extraction in time-frequency and time-space domains, which are described in the following sections.

### LSTM learning with time-frequency and time-space features of images

LSTM networks^20^, which are a special type of the recurrence neural network (RNN), are mainly applied for classifying time series or sequential data such as language modeling and speech-to-text translation^21,22^. The LSTM network was developed to solve the vanishing gradients problem encountered by the RNN. The essence of the LSTM design is the introduction of data-dependent configurations into the RNN cells to prevent the gradient of its objective function from vanishing during the data training^20^. As a result of the redesign, LSTM networks can be more robust and versatile than RNNs^23^.

As an extension of applications of the time-frequency and time-space LSTM (TF-TS LSTM) recently introduced in literature for classifying physiological signals^15^, images of histopathological tissue are vectorized into time series whose TF and TS features can be extracted for learning by the TF-TS LSTM. The extractions of the TF features that are the instantaneous frequency (IF) and spectral entropy (SE), and the TS features that are the fuzzy recurrence image entropy (FRIE) and fuzzy recurrence entropy (FRE) were described in detail in^15^. Details on how TF and TS features are used for learning by the TF-TS LSTM can also be found in^15^.

The procedure for implementing the TF-TS LSTM for image classification is as follows.

1. Vectorize an image into time series.
2. Extract IF and SE from the time series.
3. Construct the fuzzy recurrence plot (FRP) of the time series.
4. Extract FRIE and FRE of the FRP.
5. Train the LSTM with the extracted TF and TS features.

The IF of a time series, which is the average of frequencies *f* over time instant *t*, is expressed as

2 $$\begin{aligned} IF(t) = \frac{\int _{-\infty }^{\infty } f P(t,f) df}{\int _{-\infty }^{\infty } P(t,f) df}, \end{aligned}$$

where *P*(*t*, *f*) is the power spectrum.

Because Eq. (2) applies to infinitely long signal, the IF for a signal of a finite length needs to be numerically estimated. A method for estimating the IF and adopted herein was described in^15^.

The SE at time *t* is computed as

3 $$\begin{aligned} SE = - \sum _{m=1}^N p(t,m) \log _2 p(t,m). \end{aligned}$$

where the probability at time *t* and frequency *m* is

4 $$\begin{aligned} p(t,m) = \frac{P(t,m)}{\sum _f P(t,f)}, \end{aligned}$$

in which $f \in [0, fs/2]$, and *fs* is the sampling frequency.

Next, given a time series, an embedding dimension, and a time delay, the phase space of the corresponding dynamical system can be constructed and represented with a collection of vectors ${\mathbf{X}} = (\mathbf {x}_1, \dots , \mathbf {x}_N)$. Elements of an FRP are defined as^24^

5 $$\begin{aligned} FRP(i,j) = \mu (\mathbf {x}_i,\mathbf {x}_j), \, i, j = 1, \dots , N, \end{aligned}$$

where $\mu (\mathbf {x}_i,\mathbf {x}_j) \in [0, 1]$ is the fuzzy membership of similarity between $\mathbf {x}_i$ and $\mathbf {x}_j$.

By partitioning ${\mathbf{X}}$ into *c* clusters using the fuzzy *c*-means algorithm^25^, an FRP has the following three properties:

1. Reflexivity:
  6 $$\begin{aligned} \mu (\mathbf {x}_i,\mathbf {x}_i) = 1, \, i=1, \dots , N. \end{aligned}$$
2. Symmetry:
  7 $$\begin{aligned} \mu (\mathbf {x}_i,\mathbf {v}_q) = \mu (\mathbf {v}_q,\mathbf {x}_i), \, i = 1, \dots , N, q = 1, \dots , c. \end{aligned}$$
3. Transitivity:
  8 $$\begin{aligned} \mu (\mathbf {x}_i,\mathbf {x}_j) = \max [\min \{\mu (\mathbf {x}_i,\mathbf {v}_q), \mu (\mathbf {x}_j,\mathbf {v}_q)\}], q = 1, \dots , c. \end{aligned}$$

The FRIE is then computed as

9 $$\begin{aligned} FRIE = - \sum _{l=1}^L p_l \log _2 p_l, \end{aligned}$$

where *L* is the number of gray levels of the FRP (represented by the fuzzy membership grades), and $p_l$ is the probability associated with gray level *l*.

Using the concept of the entropy of a fuzzy set^26^, the FRE of an FRP is calculated as^27^

10 $$\begin{aligned} FRE = \sum _{i=}^N \sum _{j=1}^N - \mu (\mathbf {x}_i,\mathbf {x}_j) \, \log _2 \mu (\mathbf {x}_i,\mathbf {x}_j) - [1-\mu (\mathbf {x}_i,\mathbf {x}_j)] \, \log _2[1-\mu (\mathbf {x}_i,\mathbf {x}_j)]. \end{aligned}$$

## Class modeling and performance measures

### Deep-learning based class modeling

In machine learning, methods for class modeling or one-class classification^28^ aim to recognize samples of a particular class among all other class samples by focusing on the learning from a training set containing only the samples belonging to that class. This approach is different from conventional classification methods, which learn to differentiate two or more classes with training data containing samples from all the classes.

In this study, the bi-LSTM network was used for the class modeling. The network learned samples from a particular class while creating an equal number of samples for other class(es) from a single sample for each of other class(es). This design was based on the capability of the bi-LSTM network to enhance its learning from multiple copies of the same sample. Not only this design of class modeling can enhance the learning capability of the network, it can also address the problem of data imbalance often encountered in the classification of histopathological images^29^. This deep-learning approach is referred to as image-LSTM.

The network layer was specified with an output size = 100, fully connected layer = number of classes, and, while the sigmoid function is used for the LSTM gate activation, the network ends with a fully connected layer, a softmax layer, and a classification output layer. Training options of the bi-LSTM were set as optimizer = ‘Adam’ (adaptive moment estimation), including $L_2$ regularization factor, maximum number of epochs = 80 for the 2-class classification of the H&E CRC, H&E heart-failure, and IHC rectal cancer data, and 180 for the 8-class classification of the H&E CRC data, minimum batch size = 150, initial learning rate = 0.01, and gradient threshold = 1.

To compute the instantaneous frequency and spectral entropy, the sampling frequency *fs* = 300 Hz, and frequency range = [0, *fs*/2]. In this study, the value for the sampling frequency is arbitrarily chosen to extract the time-frequency features of the transformed time series of the images, but commonly used for physiological signals (https://physionet.org/challenge/2017/). To compute the Shannon entropy and fuzzy entropy of an FRP, embedding dimension = 1, time delay = 1, and number of clusters = 3. For consistent input to the bi-LSTM, the length of the sequences of the time-space features (FRIE and FRE) was designed to match with that of the time-frequency (IF and SE) sequences by segmenting the transformed time series into the number of segments being equal to the length of the time-frequency sequences. Each segment of the time series was then sequentially processed to obtain the time-space sequences.

**Figure 4:**
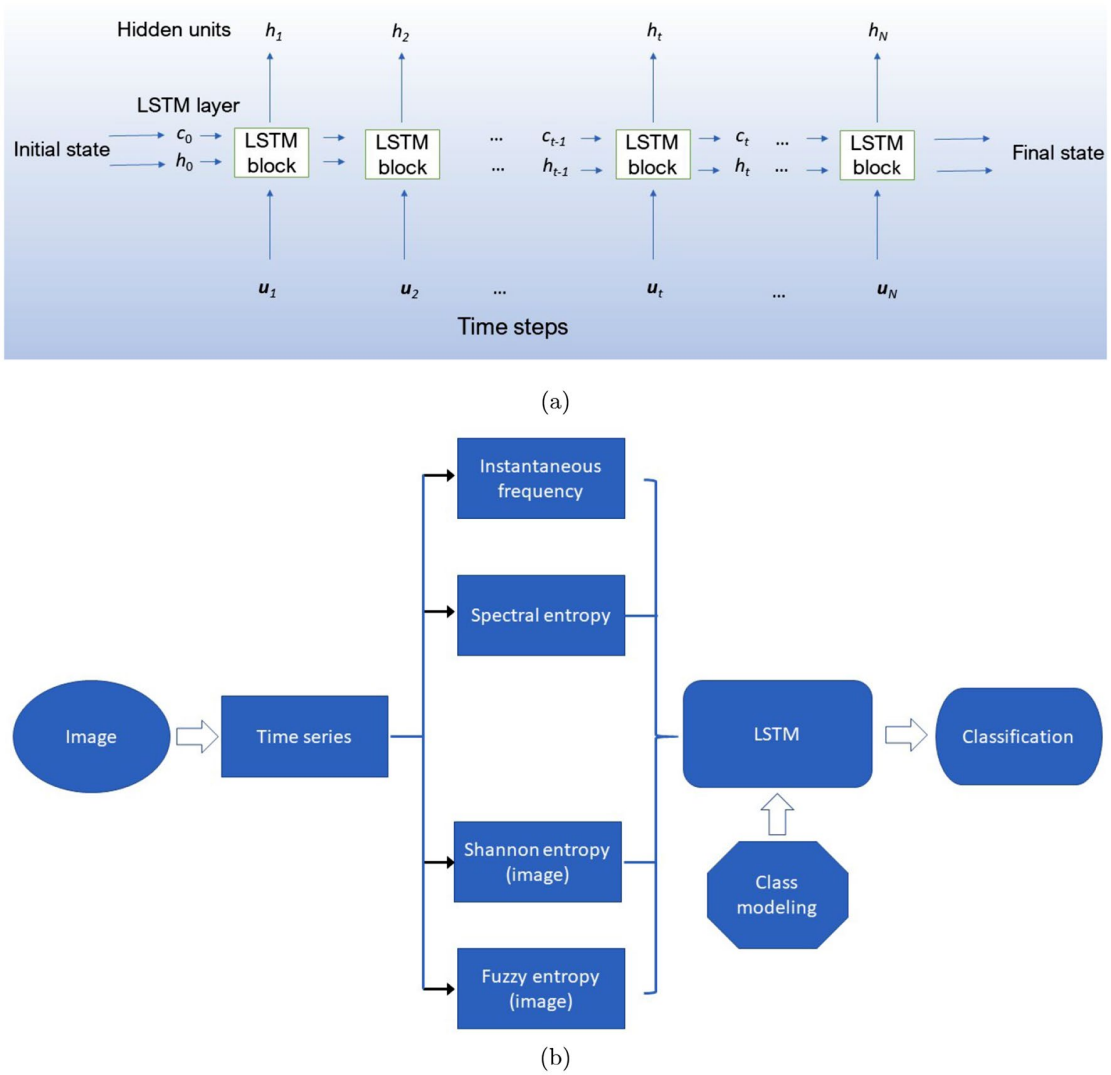
image-LSTM: (**a**) architecture, and (**b**) data pipeline.

Figure 4 shows the architecture and sequential procedure of the image-LSTM for classifying histopathological images.

### Evaluation metrics

Accuracy (ACC), sensitivity (SEN), specitivity (SPE), precision (PRE), and $F_1$ score were adopted as statistical measures of the tissue classification performance. ACC is defined as

11 $$\begin{aligned} \text {ACC} = \frac{\hbox{TP+TN}}{\hbox{P+N}}, \end{aligned}$$

where TP, TN, P, and N refer to true positive, true negative, condition positive, and condition negative, respectively. In this study, for each tissue class considered as positive, TP is the number of positive samples correctly classified as positive, TN the number of correctly classified negative samples, P the total number of positive samples, and N the total number of negative samples.

SEN is defined as

12 $$\begin{aligned} \text {SEN} = \frac{\hbox{TP}}{\hbox{P}}. \end{aligned}$$

SPE is defined as

13 $$\begin{aligned} \text {SPE} = \frac{\hbox{TN}}{\hbox{N}}. \end{aligned}$$

PRE is calculated as

14 $$\begin{aligned} \text {PRE} = \frac{\hbox{TP}}{\hbox{TP+FP}}. \end{aligned}$$

$F_1$ score is the harmonic mean of precision and sensitivity and determined as

15 $$\begin{aligned} F_1 = \frac{\hbox{2TP}}{\hbox{2TP+FP+FN}}, \end{aligned}$$

where FP is the number of negative samples incorrectly classified as positive, and FN the number of positive samples incorrectly classified as negative.

For the prediction and prognosis of DNp73 in rectal cancer, survival rates of more and less than 5 years are defined as true positive rate (TPR) and true negative rate (TNR), respectively, which are calculated as

16 $$\begin{aligned} \text {TPR}= & {} \frac{\hbox{TP}}{\hbox{P}}. \end{aligned}$$

17 $$\begin{aligned} \text {TNR}= & {} \frac{\hbox{TN}}{\hbox{N}}. \end{aligned}$$

## Results

**Figure 5:**
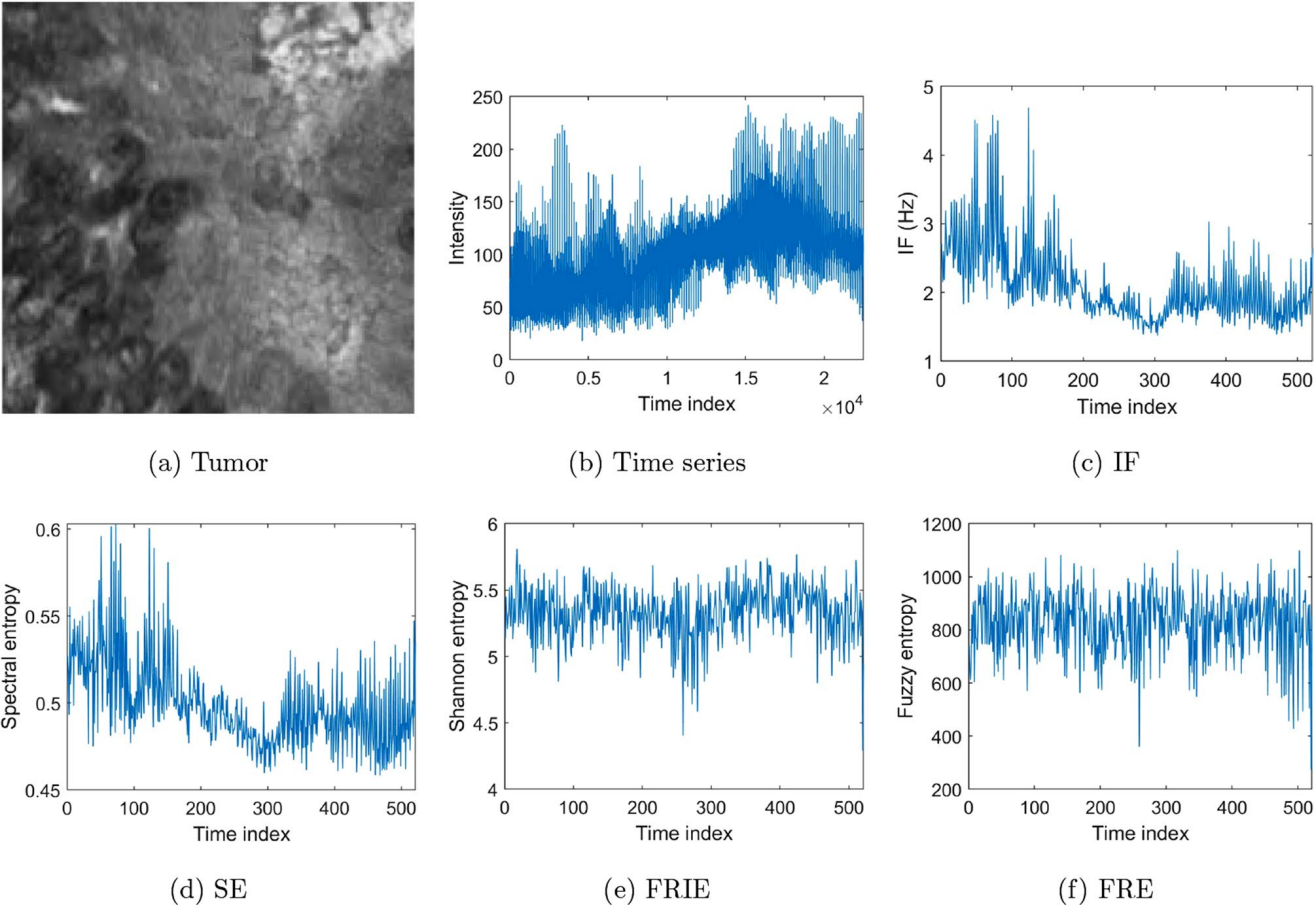
Time-frequency and time-space features extracted from time series of a grayscale image of H&E tumor tissue of colorectal cancer.

**Figure 6:**
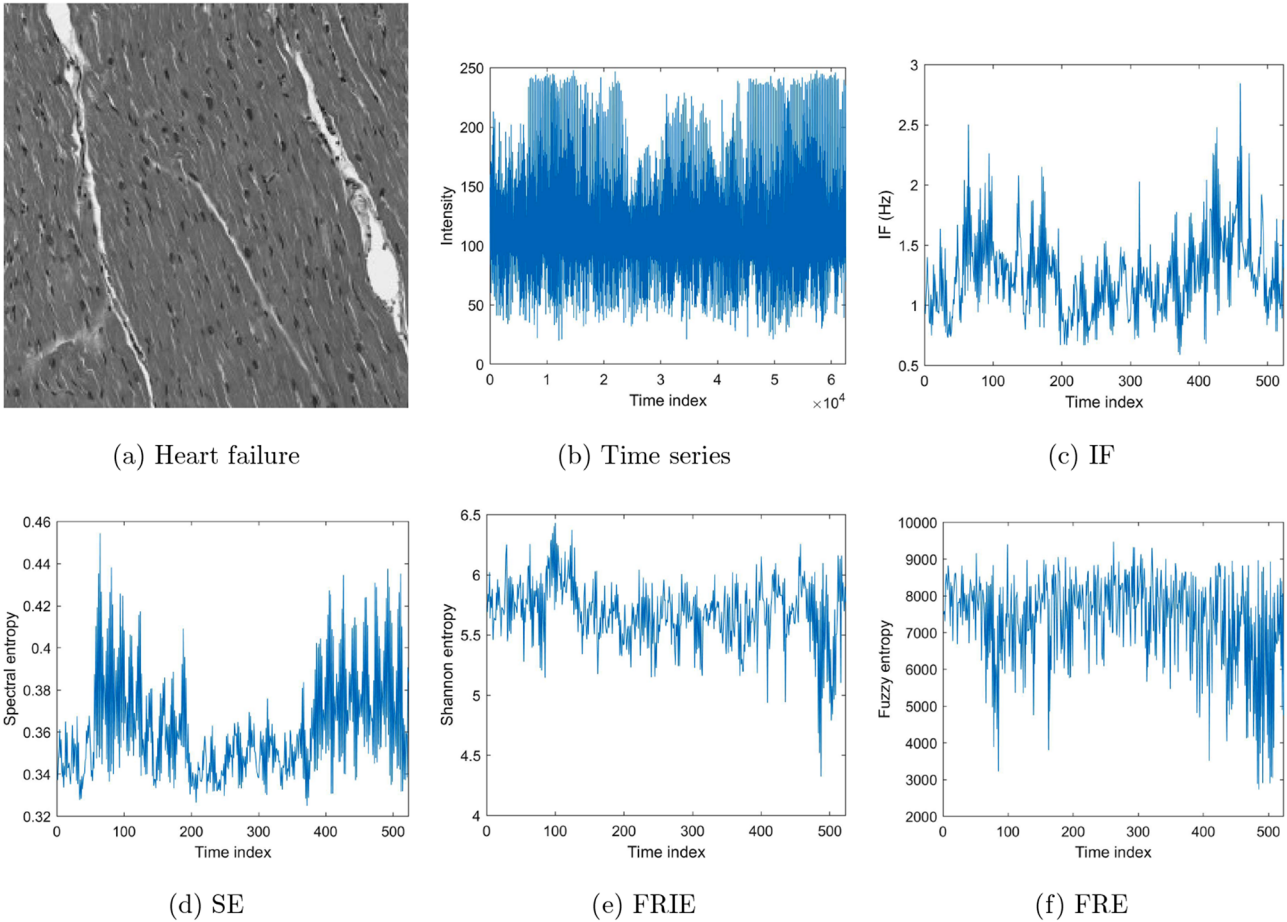
Time-frequency and time-space features extracted from time series of a grayscale image of H&E heart tissue.

**Figure 7:**
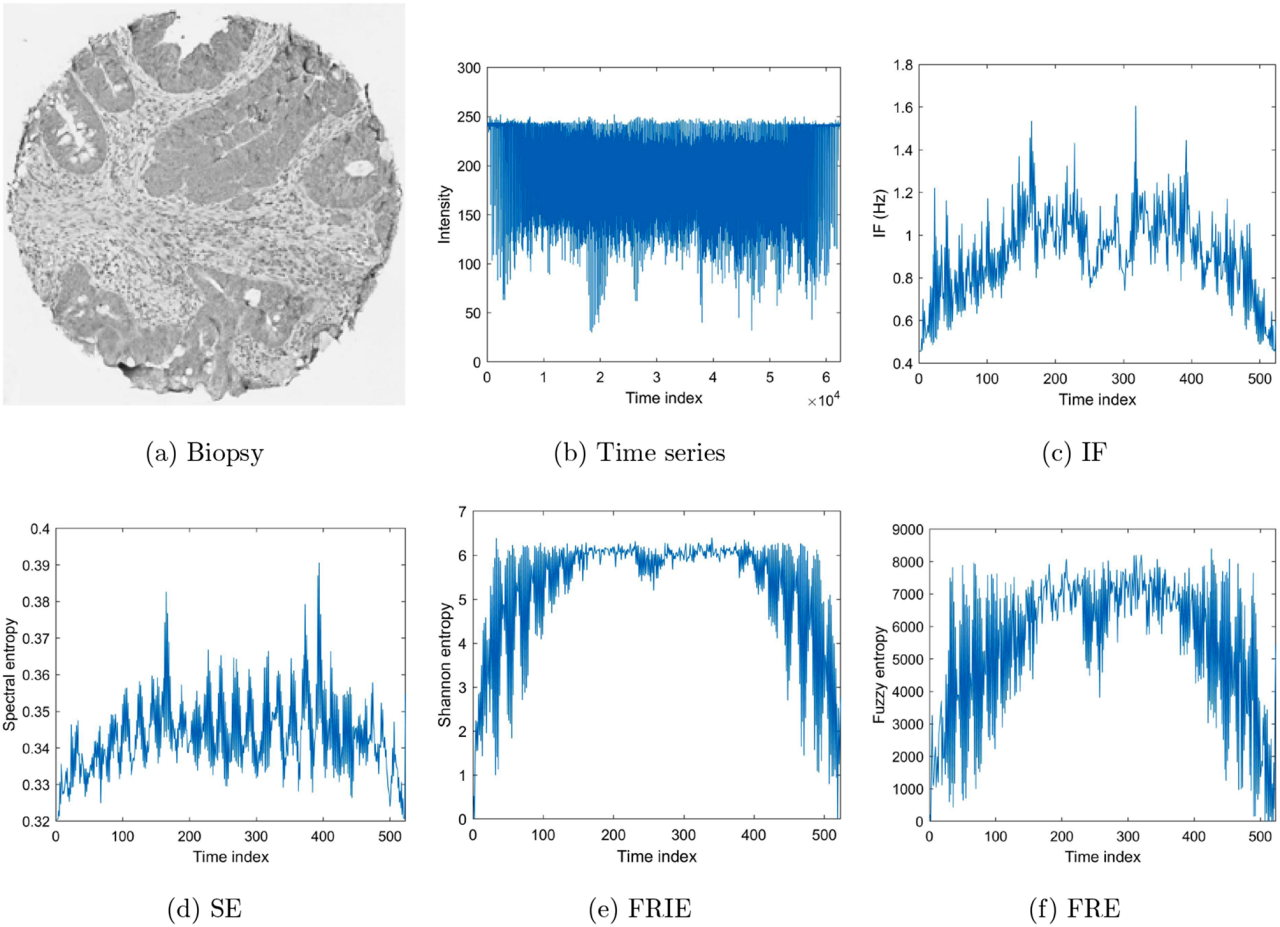
Time-frequency and time-space features extracted from time series of a grayscale image of IHC rectal-cancer tissue obtained from a patient having survival rate $\le$ 5 years.

To carry out the image data pre-processing for sequential feature extraction described earlier, because of the large size of the IHC slides of rectal cancer, these images were resized to $250 \times 250 \times 3$ pixels to reduce the time for subsequent computations and network training. Figures 5, 6, and 7 show examples of the conversions of images into time series, time-frequency features, and time-space features using H&E CRC, H&E heart-failure, and IHC rectal cancer datasets, respectively. These features of the images were combined to constitute multi-dimensional sequences and used as the input into the bi-LSTM for learning and classification.

Tables 1 and 2 show the classification results obtained from different methods using the H&E CRC and heart-failure data obtained as averages of ten runs of tenfold cross-validation (CV), H&E heart-failure data as averages of ten runs of twofold (for comparison with previously reported results) and threefold CVs (for comparison with previously reported results), and IHC rectal-cancer data as averages of ten runs of tenfold CV, respectively.

**Table 1:** Classification results.

Method	ACC (%)	SEN (%)	SPE (%)	PRE (%)	$F_1$
**H&E colorectal cancer: 2 Classes, tenfold CV**					
SVM-histogram^16^	95.70 ± n/m	n/m	n/m	n/m	n/m
SVM-LBP^16^	94.90 ± n/m	n/m	n/m	n/m	n/m
image-LSTM	100 ± 0.0	100 ± 0.0	100 ± 0.0	100 ± 0.0	1 ± 0.0
**H&E colorectal cancer: 8 Classes, tenfold CV**					
SVM-histogram^16^	80.80 ± n/m	n/a	n/a	n/a	n/a
SVM-LBP^16^	76.20 ± n/m	n/a	n/a	n/a	n/a
image-LSTM	99.96 ± 0.08	n/a	n/a	n/a	n/a
**H&E heart-failure: tenfold CV**					
image-LSTM	100 ± 0.0	100 ± 0.0	100 ± 0.0	100 ± 0.0	1 ± 0.0
**H&E heart-failure: twofold CV**					
RF^17^	86.20 ± 1.00	90.90 ± 2.00	82.30 ± 3.00	n/m	n/m
CNN^17^	93.20 ± 1.00	98.50 ± 1.00	90.00 ± 0.20	n/m	n/m
image-LSTM	99.87 ± 0.23	99.74 ± 0.46	100.00 ± 0.00	100.00 ± 0.00	0.999 ± 0.002
**H&E heart-failure: threefold CV**					
RF^17^	87.60 ± 5.00	88.10 ± 7.00	87.20 ± 4.00	n/m	n/m
CNN^17^	95.90 ± 2.00	97.10 ± 1.00	94.90 ± 5.00	n/m	n/m
image-LSTM	99.87 ± 0.15	99.73 ± 0.31	100.00 ± 0.00	100.00 ± 0.00	0.999 ± 0.002

*n/m* not mentioned, *n/a* not applicable.

**Table 2:** Prediction and prognosis of DNp73: IHC rectal-cancer data.

Method	ACC (%)	> 5 years (%)	$\le$ 5 years (%)
**Biopsy with pRT**			
ResNet101^19^	92.50 ± 12.08	100.00 ± 0.00	70.00 ± 48.30
DenseNet201^19^	92.50 ± 16.87	96.67 ± 10.54	80.00 ± 42.16
image-LSTM	100.00 ± 0.00	100.00 ± 0.00	100.00 ± 0.00
**Biopsy without pRT**			
ResNet50^19^	94.00 ± 9.66	70.00 ± 48.30	100.00 ± 0.00
VGG16^19^	94.00 ± 9.66	80.00 ± 42.16	97.50 ± 7.91
DenseNet201^19^	96.00 ± 8.43	90.00 ± 31.62	97.50 ± 7.91
image-LSTM	100.00 ± 0.00	100.00 ± 0.00	100.00 ± 0.00
**Tumor with pRT**			
ResNet101^19^	90.00 ± 16.10	92.50 ± 12.08	85.00 ± 33.74
InceptionV3^19^	93.33 ± 11.65	95.00 ± 10.54	90.00 ± 31.62
DenseNet201^19^	93.33 ± 16.10	92.50 ± 16.87	95.00 ± 15.81
NasNetLarge^19^	93.33 ± 16.10	95.00 ± 15.81	90.00 ± 21.08
image-LSTM	100.00 ± 0.00	100.00 ± 0.00	100.00 ± 0.00
**Tumor without pRT**			
GoogLeNet^19^	94.29 ± 18.07	96.00 ± 12.65	90.00 ± 31.63
ResNet50^19^	94.29 ± 12.05	96.00 ± 8.43	90.00 ± 21.08
DenseNet201^19^	94.29 ± 13.80	96.00 ± 8.43	90.00 ± 31.62
InceptionV3^19^	94.29 ± 12.05	100.00 ± 0.00	80.00 ± 42.16
ResNet101^19^	95.71 ± 9.64	98.00 ± 6.32	90.00 ± 21.08
image-LSTM	100.00 ± 0.00	100.00 ± 0.00	100.00 ± 0.00

For the 2-classification (stroma and tumor) of the H&E CRC data, the image-LSTM provided perfect results in terms of accuracy, sensitivity, specificity, precision, and $F_1$ score, where applicable, and outperformed other classification methods (support vector machines and histogram = SVM-histogram, and support vector machines and local binary patterns = SVM-LBP) using the same dataset reported in literature^16^.

Similarly, the image-LSTM outperformed the random forest (RF) and convolutional neural network (CNN) classifiers studied in^17^ in testing the H&E heart-failure dataset using twofold and threefold cross-validation procedures for evaluating the performance of classifiers.

Once again, the image-LSTM provided excellent and better results (100%) than many pre-trained convolutional neural network (CNN) models recently reported^19^ for classifying the biopsy and tumor samples with and without pRT in terms of accuracy, and survival rates > and $\le$ 5 years.

**Figure 8:**
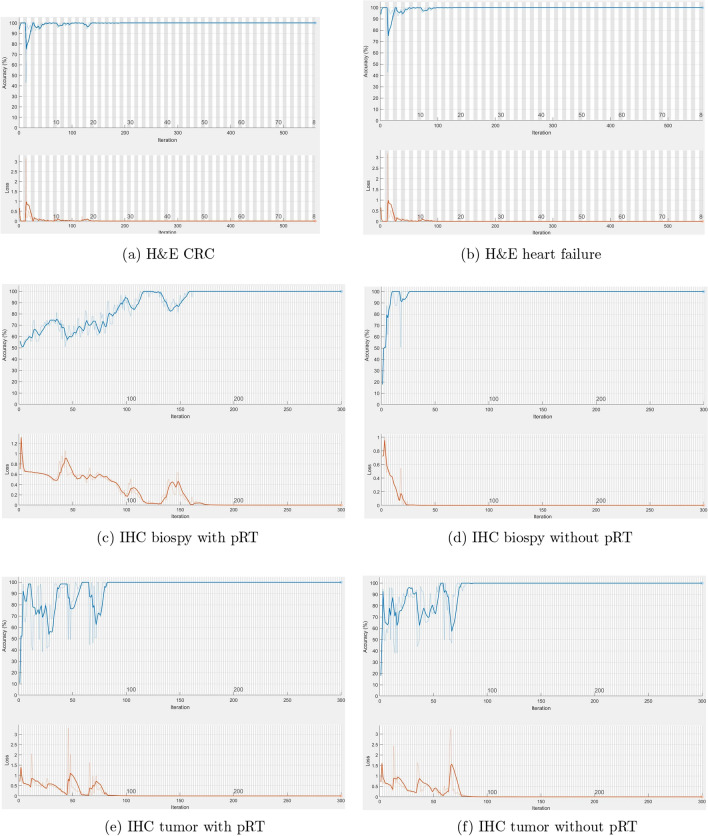
Plots of image-LSTM training progresses using different datasets.

To examine how quickly the image-LSTM accuracy was improving, and whether the network trainings could be overfitted with the training data, Fig. 8 shows the plots of the model training histories in terms of accuracy and loss using six different datasets for the tenfold CV. In general, if the loss keeps decreasing, the model could be overfitted; and when the loss becomes equal (decreasing to a point of stability), the model is considered either to be of a good fit or reaches a local minimum. By observing the plots for different datasets, the model quickly improved the accuracy and all the trainings did not result in overfitting.

## Discussion

Regarding the classification of the H&E CRC data, there are much larger differences in the average accuracy obtained from the SVM-histogram (about 15% difference) and SVM-LPB (about 20% difference) classifiers for 2-class and 8-class classifications, where the latter case is lower than the former. The average accuracy provided by the image-LSTM classifier for the 8-class classification (99.96%) is almost the same as for the 2-class classification (100%). The better performance of the image-LSTM suggests that while the sequential features of the time series transformed from the original images helped increase the differentiation of the class properties, the formation of multi-dimensional sequential features allows the enhancement of the deep learning of long-term dependencies in more than one dimension. In fact, an important task of an LSTM is to decide what information to forget and keep from the cell state, and these two things are combined to make an update to the state. The time-frequency and time-space features enabled the updating process more effective, resulting in a better classifier.

The transformation of images into time series for extracting time-frequency and time-space features and class modeling using the bi-LSTM have shown the classification design effective as the results obtained from the image-LSTM outperformed the CNN, which is also a deep-learning model and used the direct input of the images, in both twofold and threefold cross-validations.

H&E stained slides are used by pathologists for disease diagnosis and patient treatment. Images of cancer tissue are routinely examined by pathologists for cancer type identification and prognosis. The power of the precise classification of H&E stained images provided by the image-LSTM offers a useful AI tool to assist pathologists in carrying out timely analysis of large volumes of images.

The prediction and prognosis power of the protein marker DNp73 in rectal cancer can be discovered with the use of the image-LSTM for differentiating both biopsy and tumor samples of the rectal-cancer patients between with and without pRT. Recent results^19^ showed the usefulness of several pre-trained CNN models for discovering the function of DNp73 in rectal cancer. In comparison with the pre-trained CNN models, the present classification results obtained from the image-LSTM not only confirm the recent findings but also provide a better AI tool for clinical decision making and accurate forecast of the future course of the cancer under pRT.

The high performance of the image-LSTM in classifying IHC slides can be very useful for timely biomarker discovery. Current practice in pathology relies on manual scoring of protein expression mainly based on colors to assess the capability of prediction and prognosis of the candidate protein as a biomarker. The classification accuracies based on the expressions of DNp73 with pRT and without pRT suggest the power of the present AI method with an implication that if the examination of a tissue taken from a rectal cancer patient is predicted to have a short survival ($\le$ 5 years), then the clinical decision would be to recommend the patient to be treated with pRT^30^.

While traditional IHC analysis did not provide any predictive and prognostic information of DNp73 expression in the rectal cancer patients without or with pRT^19^, both predictive and prognostic power of DNp73 expression can be discovered by the current AI approach. Such a discovery is very useful for optimal treatment and clinical decision making in pRT. The AI approach reported herein is not only useful for studying the protein expression in rectal cancer patients but can also be applied for discovering biomarkers in other types of cancer. A certain advantage discovered from the findings regarding the computer implementation of the AI is that accurate classification results can be achieved without the need for the manual extraction of regions of interest from whole-slide images. Such a freedom from the manual analysis is particularly useful for guaranteeing objective and reproducible results as well as alleviating time-consuming work to help life-science researchers focus on more important aspects in their study^30^.

## Conclusion

An approach for classifying histopathological images by feeding the LSTM with sequential features extracted from time-frequency and time-space domains together with the class modeling has been presented and discussed in the foregoing sections. The key attribute is the capture of effective sequential features of texture-rich images in pathology that can leverage the power of the LSTM in learning sequential characteristics for pattern recognition. Classification results obtained from testing the new approach with both H&E and IHC image data outperformed other baseline methods. The image-LSTM appears to be a useful AI tool in the big data analysis of digital pathology for disease diagnosis, prognosis, and biomarker discovery, where the effective handling of big data can significantly contribute to modern healthcare systems^31^. The tool presented herein can be applied for classifying other types of microscope images of cells or tissues.

## Data Availability

The computer software written in MATLAB for the image-LSTM tool is publicly available at the author’s personal homepage: https://sites.google.com/view/tuan-d-pham/codes.
